# Utilization of *Oryza officinalis* Genetic Resources for Developing a New Restorer Line and a High-Yield Blast-Resistant Hybrid Rice

**DOI:** 10.3390/plants15213269

**Published:** 2026-10-23

**Authors:** Linan Zhai, Xiaowei Yan, Funeng Xing, Mingchao Zhao, Qingyu Wang, Bangji Zhou, Yong Yun, Xiaorong Xiao, Yapeng Li, Qingjie Tang

**Affiliations:** 1 Cereal Crops Institute, Hainan Academy of Agricultural Sciences/Key Laboratory of Crop Genetics and Breeding of Hainan Province, Haikou 571100, China; zhailn868@163.com (L.Z.); 13078994838@163.com (X.Y.); xingfuneng@hnaas.org.cn (F.X.); 123zhaomingchao@163.com (M.Z.); 18789169355@163.com (Q.W.); 18389302711@163.com (B.Z.); xiaorong1990829@163.com (X.X.);; 2 Sanya Institute, Hainan Academy of Agricultural Sciences, Sanya 572025, China; yunyong@hnaas.org.cn

**Keywords:** *Oryza officinalis*, exogenous DNA introduction, blast resistance, hybrid rice, yield stability

## Abstract

To overcome reproductive barriers and utilize beneficial genes from wild rice, total DNA from blast-resistant *Oryza officinalis* was introduced into elite restorer line R225 via panicle-neck injection (pollen-tube pathway), and after multi-generation selection, the novel restorer ‘Hai Hui 2011′ was developed and crossed with ‘Long Te Fu A’ to produce the hybrid ‘Te You 2011’. Multi-year field trials in Hainan showed that ‘Te You 2011’ yielded 8.48–8.70 t/ha in early seasons, with increases of 2.23–9.70% over the check ‘Te You 009’, and an 8.36% increase in late season, indicating that it has good yield potential. SSR marker RM154 detected a 183bp specific fragment shared with the wild donor in ‘Hai Hui 2011’ and *Oryza officinalis* but absent in ‘R225’, providing preliminary evidence of genetic relatedness. Marker alleles associated with four known blast resistance loci (*Pib*, *Pik*, *Pi54*, and *Pi33*) were detected in both ‘Hai Hui 2011’ and ‘Te You 2011’. Under natural field conditions and artificial inoculation with the predominant Hainan YF race group, both lines showed strong resistance. These findings preliminarily suggest that panicle-neck injection of exogenous total DNA may be a potential approach for introducing wild-rice genetic material, and that ‘Te You 2011’ is a promising high-yielding, blast-resistant hybrid for tropical and subtropical rice regions.

## 1. Introduction

Rice blast, caused by the fungal pathogen *Magnaporthe oryzae*, is one of the most destructive diseases in global rice production and poses a major threat to food security [1,2]. The widespread adoption of hybrid rice has made substantial contributions to China’s food supply, with its heterotic advantage largely determined by the genetic complementarity between parental lines and the overall agronomic performance of restorer lines. Consequently, breeding elite restorer lines is a pivotal step in developing strong heterotic combinations. However, most currently cultivated restorer lines possess limited blast resistance and a narrow genetic base of resistance sources, underscoring the urgent need to introgress novel resistant germplasm to broaden parental genetic diversity. Deploying resistant varieties remains the most economical, effective, and environmentally sound strategy for blast management [3]. Nevertheless, the high virulence variability of blast races frequently leads to the breakdown of single resistance genes within a few years of commercial cultivation, resulting in loss of varietal resistance. Therefore, the discovery of new resistance sources and the pyramiding of multiple resistance genes are essential for developing rice varieties with broad-spectrum and durable blast resistance.

Hainan Province is located in tropical southern China. Rice is cultivated on approximately 267,000 ha annually, with an average yield of about 5.5–6.0 t/ha. Both conventional and hybrid rice are grown, and widely cultivated varieties and combinations include ‘Te You 009’, ‘Te You 458’, ‘Boyou’ series, and other locally adapted hybrids. Rice production in Hainan faces several challenges, including frequent typhoons and heavy rainfall during the late season, high temperature stress, and destructive diseases such as rice blast. Among these, blast disease caused by *Magnaporthe oryzae* is a major constraint to stable yield, particularly under the warm and humid tropical conditions of the province. These challenges underscore the need for developing rice varieties with broad-spectrum blast resistance and good adaptability to the local environment.

*Oryza officinalis* Wall. ex G. Watt, a wild relative of cultivated rice (*Oryza sativa*), has accumulated a wealth of favorable traits—including disease resistance and stress tolerance—through long-term natural selection, making it a valuable germplasm resource for modern rice genetic improvement [4]. This species is widely distributed across tropical and subtropical regions of Asia, Africa, and Australia [5], and within China, it is predominantly found in Yunnan, Guangdong, Guangxi, and Hainan provinces. However, severe reproductive isolation between *O. officinalis* and cultivated rice largely precludes the direct utilization of its beneficial genes through conventional sexual hybridization.

To circumvent this barrier, the exogenous total DNA introduction technique—also referred to as the pollen-tube pathway or panicle-neck injection method—was developed. Originally proposed by Zhou Guangyu based on the DNA fragment hybridization hypothesis [6], this approach enables the creation of heritable variations by introducing donor total DNA fragments into recipient plants [7]. Two main implementation methods are commonly used: the stigma-dropping method and the panicle-stem injection method. In the stigma-dropping method, flowers that have opened on the day of treatment are selected; 1–3 h after flowering, the stigmas are cut off, and exogenous DNA solution is applied to the exposed ovaries, after which the glumes are closed and the panicles are bagged. In the panicle-stem injection method, the main panicle, with a distance of 1–5 cm between the flag leaf auricle and the second leaf auricle, is selected, and the exogenous DNA solution is injected into the first internode below the panicle neck using a microsyringe, followed by bagging and seed harvesting at maturity. The underlying principle of these methods is that, after pollination, exogenous DNA can penetrate along the pollen-tube pathway, pass through the pit fields or perforations of the nucellar cell walls, enter the embryo sac, and then transform the egg cells, zygotes, or early embryonic cells—which lack normal cell walls and thus act as natural protoplasts—facilitating DNA uptake. The active DNA replication following fertilization further promotes integration of the exogenous fragments.

This technique has been successfully applied in breeding programs for multiple crops, including wheat [8], cotton [9], maize [10], and rice [11], thereby providing a practical means of harnessing desirable genes from wild relatives. For instance, previous studies have employed panicle-neck injection to introduce DNA from *Oryza meyeriana* Baill into rice from Hainan, yielding stable variants with exogenous DNA integration verified by SSR marker analysis [12].

Identification and pyramiding of resistance genes are central to blast resistance breeding. To date, over 100 resistance genes and approximately 500 quantitative trait loci (QTLs) have been identified in rice [13], among which *Pib* [14], *Pik* [15], and *Pi54* [16,17] are recognized as broad-spectrum or durable resistance genes. Notably, *Pib* and *Pik* confer strong resistance against the predominant *Magnaporthe oryzae* races (primarily ZB and ZC groups) prevalent in South China [18]. *Pi54*, originally designated as *Pi-kh*, was identified from the indica cultivar Tetep and exhibits broad-spectrum resistance against multiple blast isolates from various countries [19]. Meanwhile, *Pi33* enhances the recognition of specific pathogen races, thereby contributing to race-specific resistance [20]. The pyramiding of multiple resistance genes can substantially broaden the resistance spectrum and mitigate the risk of resistance breakdown caused by race-specific pathogen adaptation [21]. At present, multiple rice blast resistance genes such as *Pia* [22], *Pib* [23], *Pi33* [20], *Pid2* [24], *Pik* [25], *Pigm* [26], *Pi21* [27], *Pi9* [28], *Pi20* [29,30], *Pi54* [31], etc., have been developed as functional or specific molecular markers or target gene alleles, laying the foundation for accurate identification of rice germplasm resources for blast resistance genotypes and molecular marker assisted disease resistance breeding.

In this study, the highly blast-resistant *Oryza officinalis* from Hainan served as the donor, while the elite *indica* restorer line ‘R225’—characterized by desirable agronomic traits but moderate susceptibility to blast—was used as the recipient. Through panicle-neck injection of total DNA from the wild donor, combined with multi-year and multi-generation phenotypic screening, we successfully developed the novel restorer line ‘Hai Hui 2011’. Subsequently, this restorer line was crossed with the widely utilized three-line sterile line ‘Long Te Fu A’ to generate the hybrid combination ‘Te You 2011’. The specific objectives of this study were: (1) to characterize the key agronomic traits, yield performance, and blast resistance levels of both ‘Hai Hui 2011’ and ‘Te You 2011’; (2) to conduct a preliminary investigation into the genetic relatedness between ‘Hai Hui 2011’ and *O. officinalis* using SSR markers; (3) to explore the composition of blast resistance genes in these materials. Our findings are expected to provide both a theoretical foundation and valuable parental germplasm resources for utilizing wild rice species in disease resistance breeding and parental line innovation in hybrid rice.

## 2. Results

### 2.1. Agronomic Traits of the Restorer Line ‘Hai Hui 2011’ and Hybrid ‘Te You 2011’

‘Hai Hui 2011’ had a growth duration of approximately 125 days in early rice and 120 days in late rice in Hainan. It exhibited a moderate plant type, vigorous growth, and strong stress tolerance. The line produced abundant pollen, showed high fertility, and had a uniform population with favorable late-stage maturity (Figure 1 and Figure 2). Major agronomic parameters were as follows: plant height 110.5 cm, panicle number 7.3, panicle length 21.3 cm, 140.5 grains per panicle, 85.5% seed setting rate, and 25.3 g thousand-grain weight (Table 1). Its abundant pollen production coupled with high seed setting rate significantly enhanced hybrid seed yield and stability. Therefore, this line serves as an excellent male parent for hybrid rice production.

The hybrid ‘Te You 2011’ had a growth duration of 125–130 days in the early rice season, which was 0–4 days shorter than that of the control variety ‘Te You 009’ (the widely grown hybrid ‘Te You 009’ as the control). In the late rice season, its growth duration was 115.3 days, 2.4 days shorter than the control. The major agronomic traits of ‘Te You 2011’ were as follows: plant height 108.8 cm, panicle number 6.2, panicle length 22.2 cm, 153.3 grains per panicle, 89.8% seed setting rate, and 28.6 g thousand-grain weight (Figure 3, Table 1).

Seeds of the original restorer line R225, the new restorer line ‘Hai Hui 2011’, the maternal parent ‘Long Te Fu A’, and the hybrid rice ‘Te You 2011’ were photographed. As shown in Figure 4, R225 exhibited a medium grain shape, whereas ‘Hai Hui 2011’, a new restorer line developed by introgression of genes from *Oryza officinalis*, had longer grains. ‘Long Te Fu A’, the maternal parent of ‘Te You 2011’, had short, round grains. The grain shape of ‘Te You 2011’ generally resembled that of its maternal parent, although its grain length was somewhat greater than that of ‘Long Te Fu A’. These observations suggest that the grain shape of ‘Te You 2011’ was strongly influenced by its maternal parent, and that the altered grain shape of ‘Hai Hui 2011’ was also reflected to some extent in the hybrid progeny.

### 2.2. Yield Performance of ‘Te You 2011’

The variety showed some degree of average yield advantage in multiple yield trials across different years and seasons. Yield data for each site were presented as means ± standard error (SE) of three replications. Differences among varieties were assessed using combined analysis of variance (ANOVA), with multiple comparisons performed by the least significant difference (LSD) test. Asterisks indicate significance levels: * *p* < 0.05 and ** *p* < 0.01. The results were as follows.

In the 2020 early-season regional trial in Hainan (Table 2), the mean yield of ‘Te You 2011’ was 8.57 ± 0.64 t/ha, compared with 8.07 ± 0.58 t/ha for the control ‘Te You 009’, representing an average increase of 6.20%. The difference was highly significant (*p* < 0.01, ∗∗). The daily yield was 68.70 kg/ha/day, and 100% of the trial locations showed positive yield responses. These results indicate that the variety had a clear average yield advantage in the 2020 early-season regional trial.

In the 2021 early-season continued regional trial (Table 2), the mean yield of ‘Te You 2011’ was 8.70 ± 0.72 t/ha, compared with 8.51 ± 0.66 for the control, an average increase of 2.23%; however, the difference was not statistically significant. Among the six sites, Chengmai, Sanya, and Qionghai showed yield increases, whereas Tunchang, Danzhou, and Lingshui showed negative responses, with changes of −5.48%, −1.81%, and −1.08%, respectively. These negative responses may be related to site-specific environmental conditions, soil fertility, disease and pest pressure, but the overall mean yield remained higher than that of the control. The daily yield was 69.90 kg/ha/day.

In the 2021 production trial (Table 3), the average yield was 8.48 ± 0.66 t/ha, compared with 7.73 ± 0.60 t/ha for the control, an average increase of 9.70%, but the difference did not reach a significant level (*p* > 0.05). Site-to-site variation was large: Tunchang showed a decrease of 6.67%, whereas the other sites showed increases, with Lingshui increasing by 25.23%. This variation may reflect environmental differences among sites; nevertheless, the overall effect was a positive yield increase.

In the late rice season of 2024, an expanded-season independent trial (Table 4) recorded an average yield of 5.96 ± 0.23 t/ha for ‘Te You 2011’, compared with 5.50 ± 0.27 t/ha for the control during the same season, representing an 8.36% increase, with a daily yield of 51.60 kg/ha/day; the difference was significant (*p* < 0.05, *). Among the seven sites, only Wenchang showed a slight negative response (−0.50%), while the remaining sites showed yield increases. The lower mean yield in the late season than in the early season may be related to seasonal temperature and light conditions and to frequent typhoons and rainfall during the late season in Hainan.

Across the multiple trials conducted over three years, the average yield of ‘Te You 2011’ consistently exceeded that of the control. Early-season mean yields ranged from 8.48 to 8.70 t/ha. Yield increases ranged from 2.23% to 6.20% in the regional trials and from 8.36% to 9.70% in the production and independent trials. Negative yield responses occurred at some sites, indicating that yield performance may be affected by environmental conditions; however, the overall effect was a positive yield increase. These results suggest that the variety has high yield potential and warrants further testing and demonstration in Hainan and similar tropical rice-growing areas.

### 2.3. Blast Resistance of Restorer Line ‘Hai Hui 2011’ and Hybrid ‘Te You 2011’

Under multi-year, multi-location field conditions from 2020 to 2024, neither the restorer line ‘Hai Hui 2011’ nor its derived hybrid rice ‘Te You 2011’ developed neck blast or leaf blast. In artificial inoculation assays, both ‘Hai Hui 2011’ and ‘Te You 2011’ exhibited a leaf blast resistance rating of 2 (resistant), whereas the control ‘Te You 009’ showed a rating of 8 (highly susceptible). These results indicated that ‘Hai Hui 2011’ and its hybrid ‘Te You 2011’ possess excellent resistance to the dominant populations of Magnaporthe oryzae in Hainan.

As shown in Figure 5, ‘Te You 2011’ amplified bands associated with *Pib* (365 bp), *Pik* (176 bp), *Pi54* (216 bp), and *Pi33* (198 bp). ‘Long Te Fu A’ amplified *Pib*, *Pi54*, and *Pi33* but not *Pik*. ‘Hai Hui 2011’ amplified all four markers. ‘R225’ amplified only *Pik* and *Pi33*, whereas *O. officinalis* amplified only *Pib* and *Pi54*. ‘Hai Hui 2011’ was developed by introducing Hainan *O. officinalis* DNA into ‘R225’ via panicle-neck injection followed by pedigree selection.

These results suggested that the *Pik* marker in ‘Te You 2011’ was most likely inherited from the paternal parent, as it was absent in ‘Long Te Fu A’ but present in both ‘Hai Hui 2011’ and ‘R225’. *Pi33* was present in ‘R225’, ‘Long Te Fu A’, ‘Hai Hui 2011’, and ‘Te You 2011’ but absent in *O. officinalis*, suggesting a parental rather than donor origin. *Pib* and *Pi54* were absent in ‘R225’ but present in *O. officinalis*, ‘Long Te Fu A’, ‘Hai Hui 2011’, and ‘Te You 2011’. Therefore, the newly detected *Pib* and *Pi54* markers in ‘Hai Hui 2011’ were consistent with the markers that might have been introduced from *O. officinalis*, although the maternal parent also carried these markers.

Previously, ‘R225’ was classified as moderately susceptible, whereas ‘Hai Hui 2011’ and ‘Te You 2011’ had an artificial inoculation score of 2 (resistant). This phenotypic improvement was consistent with the additional *Pib* and *Pi54* markers in ‘Hai Hui 2011’; however, the presence of molecular markers alone did not demonstrate gene function or a causal contribution to resistance. Further validation, including allele sequencing, expression analysis, genetic segregation analysis, and inoculation tests, will be required. Overall, ‘Hai Hui 2011’ and ‘Te You 2011’ showed good blast resistance under the tested conditions and carried multiple blast-resistance-associated markers, making them candidate materials for breeding blast-resistant hybrid rice in southern China.

### 2.4. Preliminary Exploration of the Genetic Relationship Between Restorer Line ‘Hai Hui 2011’ and O. officinalis

To determine whether the restorer line ‘Hai Hui 2011’ carries genetic components derived from the donor *O. officinalis*, multiple SSR markers were used to detect polymorphisms among ‘Hai Hui 2011’, the donor material (*O. officinalis* collected from Baoting, Hainan), and the control restorer line ‘R225’. Among the markers tested, SSR marker RM154 (forward primer: ACCCTCTCCGCCTCGCCTCCTC; reverse primer: CTCCTCCTCCTGCGACCGCTCC) generated stable and distinctive polymorphic bands among the three materials. As shown in Figure 6, a specific DNA fragment of 183 bp was shared by ‘Hai Hui 2011’ and *O. officinalis* but was absent in the control line ‘R225’. These results led us to speculate that genetic segments from Oryza officinalis might have been integrated into the genome of ‘Hai Hui 2011’ and that these segments might have been associated with blast resistance. This finding suggested that the pollen-tube pathway method has potential application value for improving cultivated rice traits using genes from tropical wild rice. Of course, this was only a preliminary exploration. If proven, more experiments will be needed to verify it in the future.

## 3. Discussion

In this study, exogenous total DNA from *Oryza officinalis* was introduced into the elite restorer line ‘R225’ via panicle-neck injection. Through multi-generation directed selection, we obtained a novel, stably inherited restorer line, ‘Hai Hui 2011’. Compared with its recipient parent R225, ‘Hai Hui 2011’ showed an elongated grain shape (increased grain length) and enhanced blast resistance, indicating that some genetic material of *O. officinalis* might have been introduced into ‘Hai Hui 2011’. The hybrid ‘Te You 2011’, derived from crossing this restorer with the sterile line ‘Long Te Fu A’, exhibited positive yield responses across multiple years and trials, together with strong blast resistance. SSR markers provided preliminary evidence that *O. officinalis*-specific DNA fragments might have been integrated into the genome of ‘Hai Hui 2011’, and that this line harbored multiple blast resistance genes.

### 3.1. Exogenous Total DNA Introduction as an Effective Approach for Utilizing Beneficial Genes from Wild Rice

Reproductive isolation in distant hybridization is a major barrier to exploiting wild rice resources. The panicle-neck injection method used in this study does not depend on gamete fusion. Instead, it utilizes the pollen tube formed after pollination to deliver donor total DNA fragments directly into the recipient embryo sac, where the plant’s own DNA repair and recombination mechanisms facilitate integration of the exogenous fragments, generating heritable variations [32,33]. This technique is operationally simple, cost- and time-effective, and does not require protoplast preparation or cell culture regeneration. It has been successfully applied in various crops: for example, introduction of the resistance gene *NPR1* and insect-resistance gene *Cry1Ab13-1* into maize conferred high resistance to both disease and insects [34]; introduction of *SlAMS* into tomato (*Solanum lycopersicum*) produced a male sterile line [35]; and introduction of *CfJMT* into petunia (*Petunia hybrida*) generated a small-leaf phenotype [36]. Zhang (2015) [37] used the pollen-tube pathway to introduce whole-genome DNA from *Fargesia spathacea* (a bamboo species) into six rice varieties and obtained high-yielding lines. In the present study, SSR marker RM154 amplified a fragment of 183 bp in ‘Hai Hui 2011’ that was identical to that in the donor *O. officinalis* but absent in the recipient ‘R225’, providing evidence that a DNA fragment from *O. officinalis* may have been integrated into the genome of ‘Hai Hui 2011’. This finding was consistent with the results of Tang et al. (2015) [12], who preliminarily verified the genetic relatedness of progeny derived from Oryza meyeriana Baill DNA introduction using SSR markers. Notably, the introduced exogenous fragments are not whole genomes but randomly integrated DNA segments; therefore, further in-depth research will be needed in the future.

### 3.2. Agronomic Traits and Yield Performance of ‘Te You 2011’

Yield is a critical determinant of the commercial viability of hybrid rice. In this study, ‘Te You 2011’ showed a certain average yield advantage in multi-year, multi-site regional trials in Hainan Province. In the early rice season of 2020, it achieved an average yield of 8.57 t/ha, a 6.20% increase over the control ‘Te You 009’ (*p* < 0.01), with 100% of trial locations showing positive yield responses. In the 2021 early-season follow-up trial, the yield further increased to 8.70 t/ha, 2.23% higher than the control, but the difference was not significant, and three of six sites showed negative responses. In the 2021 production trial, the mean yield was 8.48 t/ha, 9.70% higher than the control, and the 2024 late-season independent trial showed 8.36% higher than the control (*p* < 0.05). The control variety ‘Te You 009’, a standard check in the South China early-season indica high-yield group, has a regional trial average yield of approximately 7.42–8.09 t/ha and a growth duration of about 125 days when grown as early-season rice in South China. Compared with ‘Te You 009’, ‘Te You 2011’ showed a positive average yield increase in most trials.

Notably, negative yield responses occurred at Tunchang, Danzhou, and Lingshui in the 2021 regional trial and at Tunchang in the production trial, and the absolute yield in the late season (5.96 t/ha) was lower than that in the early season, suggesting that the variety may be sensitive to certain environments, and the lower late-season yield may be related to seasonal temperature and light conditions and frequent typhoons and rainfall in Hainan. Compared with other recently reported hybrid combinations, such as ‘Jing Liang You 1322’ (early-season yield 7.95–8.05 t/ha, yield increase 3.00–6.36% in Hainan regional trials), ‘Te You 2011’ exhibited both higher absolute yield and greater yield increases. However, because the trial years, locations, and designs differ among varieties, direct comparisons should be made with caution. Overall, ‘Te You 2011’ has high yield potential and application prospects in Hainan and similar tropical rice-growing areas, but its yield stability, broad adaptability, and extension value still require further validation through more comprehensive multi-environment trials and stability analysis.

### 3.3. Blast Resistance of ‘Hai Hui 2011’ and ‘Te You 2011’ and Its Genetic Basis

Under natural field conditions from 2020 to 2024, neither ‘Hai hui 2011’ nor ‘Te You 2011’ showed leaf blast or panicle-neck blast symptoms. In artificial inoculation tests, both ‘Hai Hui 2011’ and ‘Te You 2011’ showed leaf blast resistance grade 2 (resistant), whereas the control ‘Te You 009’ showed grade 8 (highly susceptible). These results indicated that ‘Hai Hui 2011’ and ‘Te You 2011’ possess strong resistance to the predominant Hainan blast population YF under the tested conditions.

Molecular marker analysis revealed that both ‘Hai Hui 2011’ and ‘Te You 2011’ carry marker alleles associated with four known blast resistance genes: *Pib*, *Pik*, *Pi54*, and *Pi33*. *Pib* and *Pik* were classic broad-spectrum resistance genes that confer strong resistance against the predominant blast races prevalent in South China [18]. Studies have shown that *Pib* has a frequency of 0.37 in commonly used indica parents in South China, making it one of the more widely distributed resistance genes [38]. *Pi54* (formerly *Pi-kh*), originally identified from the *indica* variety Tetep, was a broad-spectrum resistance gene that confers resistance against blast isolates from multiple countries and was relatively frequent in the mini-core collection of Chinese rice germplasm, indicating its high breeding value [39,40]. *Pi33* enhanced the recognition of specific pathogen races [20]. The pyramiding of these four genes is expected to create a complementary, multi-layered resistance barrier that broadens the resistance spectrum and reduces the risk of race-specific breakdown. Yan et al. [38] demonstrated that rice lines carrying the gene combinations *Pib* + *Pi54* + *Pi-km* or *Pi-ta* + *Pib* + *Pi54* exhibited significantly higher resistance levels than lines with single or double genes, confirming the critical role of gene pyramiding in enhancing resistance. However, the detection of marker alleles associated with *Pib*, *Pik*, *Pi54*, and *Pi33* in ‘Hai Hui 2011’ suggested that these loci might have contributed to its resistance, but functional validation and epistasis analysis will be required to determine whether these genes act additively, synergistically, or independently.

Parental genotyping was performed to clarify the origin of these marker alleles. The recipient parent R225, the donor *O. officinalis*, the female parent ‘Long Te Fu A’, the restorer parent ‘Hai Hui 2011’, and the hybrid ‘Te You 2011’ were genotyped for the four markers. The results showed that *O. officinalis* amplified the expected fragments for *Pib* and *Pi54*, whereas the recipient parent R225 did not amplify either of these two fragments. The new restorer line ‘Hai Hui 2011’ amplified all four fragments: *Pib*, *Pik*, *Pi54*, and *Pi33*. The hybrid ‘Te You 2011’ showed the same four fragments as its restorer parent ‘Hai Hui 2011’. In addition to these molecular results, ‘Hai Hui 2011’ exhibited an elongated grain shape (increased grain length) compared with its recipient parent R225, and showed enhanced blast resistance. These phenotypic changes further suggested the certain genetic genes of *O. officinalis* might been introduced into new restorer lines. However, grain shape and disease resistance can also be affected by background mutations, somaclonal variation, or selection during breeding. The marker patterns indicate that *Pib* and *Pi54* in ‘Hai Hui 2011’ may have been derived from the donor *O. officinalis*, because these fragments were present in the donor but absent in the recipient parent R225. Of course, this was not a certainty, and further verification needs to be conducted through sequencing and other methods.

SSR marker RM154 confirmed the presence of a donor-specific fragment may be shared between ‘Hai Hui 2011’ and *O. officinalis*, but the specific gene(s) or regulatory element(s) within this fragment remain unknown. Future studies will employ fine mapping or map-based cloning to characterize this fragment and determine whether it contains novel resistance-associated genes or regulatory elements.

## 4. Materials and Methods

### 4.1. Experimental Materials

The plant materials used in this study included *Oryza officinalis*, the rice restorer line ‘R225’, and the rice sterile line ‘Long Te Fu A’. The *O. officinalis* accession, previously identified as highly resistant to blast, was collected from Baoting County, Hainan Province, and is currently maintained in the Hainan Wild Rice Germplasm Repository. The restorer line ‘R225’ is an elite indica restorer line preserved in our laboratory, characterized by desirable agronomic traits and good combining ability, but moderately susceptible to blast. The sterile line ‘Long Te Fu A’ is a three-line sterile line developed by the Zhangzhou Agricultural Research Institute in Fujian Province. It exhibits strong tillering ability, excellent combining ability, and high seed production yield, and is currently one of the most widely deployed sterile lines in the rice-growing regions of South China.

### 4.2. Development of the Novel Restorer Line and Hybrid Combination

In the early rice season of January 2013, the restorer line ‘R225’ was planted at the Yongfa Experimental Station of the Hainan Academy of Agricultural Sciences in Chengmai County, Hainan Province. At the heading stage, the main panicles with a distance of 1–5 cm between the flag leaf auricle and the second leaf auricle were selected. A total of 50 µL of exogenous total DNA solution (300 µg/mL) extracted from *O. officinalis* was injected into the first internode below the panicle neck using a microsyringe, with the needle tip inserted at a downward angle of approximately 30° relative to the panicle neck node. Before flowering, the injected plants were bagged to prevent cross-pollination, and mature seeds were subsequently harvested. These seeds, designated as the first-generation variant progeny (F_1_), yielded a total of 18 grains.

In the late rice season of August 2013, all 18 F_1_ seeds were sown. At the heading stage of the F_1_ plants, we began to evaluate their morphological traits, disease incidence, and yield performance. Ten superior plants were selected, and their seeds were harvested as the F_2_ generation.

In the early rice season of January 2014, the F_2_ population was planted. From this stage onward, phenotypic evaluation was combined with marker-assisted selection to screen for lines with enhanced blast resistance and high yield. After continuous selection through multiple generations, a stable line was obtained by December 2017 and was officially designated as ‘Hai Hui 2011’.

Subsequently, in December 2017, ‘Hai Hui 2011’ was crossed as the male parent with the sterile line ‘Long Te Fu A’ (as the female parent) at flowering stage. The resulting hybrid seeds were designated as ‘Te You 2011’ (Figure 7).

### 4.3. Regional, Production, and Expanded-Season Trials of the Hybrid Rice ‘Te You 2011’

Regional trials were conducted at six locations in Hainan Province, China: Chengmai, Sanya, Tunchang, Qionghai, Danzhou, and Lingshui. The expanded-season trial was conducted at seven locations: Qionghai, Wenchang, Ding’an, Danzhou, Changjiang, Wuzhishan, and Sanya. For both the regional and expanded-season trials, a randomized complete block design with three replications was used at each location, and each plot had an area of 300 m^2^. The production trial was conducted at the same six locations as the regional trial. A randomized complete block design with three replications was also used, and each plot had an area of 667 m^2^. In all trials, the control variety was ‘Te You 009’. The control and test varieties were sown and transplanted at the same time. Crop management included pest control only, with no disease control. Seedling growth surveys, sampling, and trait assessments followed the <Technical Operating Procedures for Regional and Production Trials of Rice Varieties in Hainan Province>.

Yield data were analyzed using a mixed-model combined analysis of variance, and multiple comparisons among varieties were performed using the least significant difference (LSD) test. Disease resistance was evaluated based on natural infection under field conditions at the experimental sites and on artificial inoculation results provided by the Institute of Plant Protection, Hainan Academy of Agricultural Sciences.

The following was the artificial inoculation method for rice leaf blight. Seeds of ‘Hai Hui 2011’, ‘Te You 2011’, and the control ‘Te You 009’ were sown on leveled seedling beds. Plots measuring 30 cm × 30 cm were established, with one variety sown per plot. For each variety, 20–30 germinated seeds were sown. When seedlings reached the four-leaf stage after 14 days of growth, a conidial suspension (2 × 10^4^–5 × 10^4^ conidia/mL) of the dominant YF race group of *Magnaporthe oryzae* in Hainan was prepared and applied by spray inoculation. Each variety was tested in triplicate. After inoculation, the seedlings were covered with a black plastic film to maintain humidity at 26–28 °C for 24 h, after which the film was removed. Disease incidence was investigated 5–7 days after inoculation. The resistance/susceptibility and disease rating were evaluated according to the grading standards for seedling blast in the <Technical Regulations for Identification and Evaluation of Rice Blast Resistance in Rice Variety Trials > (Agricultural Industry Standard of the People’s Republic of China, NY/T 2646-2014).

### 4.4. Genomic DNA Extraction

Total genomic DNA was extracted from leaf tissues of *O. officinalis*, the restorer line ‘R225’, ‘Hai Hui 2011’, ‘Long Te Fu A’ and the hybrid ‘Te You 2011’. Leaf samples were ground to a fine powder under liquid nitrogen, and genomic DNA was subsequently isolated using the High-Efficiency Plant Genomic DNA Extraction Kit (spin-column format, Tiangen Biotech Co., Ltd., Beijing, China). The concentration and quality of the extracted DNA were assessed using a NanoDrop 1000 spectrophotometer and agarose gel electrophoresis.

### 4.5. Detection of Blast Resistance Genes

We used SSR markers (*Pia*, *Pib*, *Pi33*, *Pid2*, *Pik*, *Pigm*, *Pi21*, *Pi9*, *Pi20*, *Pi54*) that have already been developed as functional or specific molecular markers or target genes to detect the rice blast resistance genes of “Hai Hui 2011”, “Te You 2011”, “Long Te Fu A”, “Medicinal Wild Rice”, and “R225”(Table 5). PCR was performed in a total volume of 20 µL, containing 4 µL of rice leaf DNA, 10 µL of 2× Taq PCR Master Mix II, 2 µL of each primer (10 µmol L^−1^), and 2 µL of ddH_2_O. The amplification program was as follows: initial denaturation at 94 °C for 5 min; followed by 34 cycles of denaturation at 94 °C for 45 s, annealing at 45–60 °C for 45 s, and extension at 72 °C for 1 min; with a final extension at 72 °C for 8 min; and held at 4 °C. The amplified products were separated by electrophoresis on 2% agarose gels and photographed using a gel documentation system.

### 4.6. Genetic Relatedness Preliminary Analysis Between O. officinalis and ‘Hai Hui 2011’

To preliminarily investigate the genetic relatedness between ‘Hai Hui 2011’ and the donor *O. officinalis* [12], SSR marker analysis was performed using 33 primer pairs selected from publicly available rice SSR markers (Table 6). The PCR amplification protocol was as follows: initial denaturation at 95 °C for 5 min; followed by 35 cycles of denaturation at 95 °C for 30 s, annealing at 55 °C for 30 s, and extension at 72 °C for 50 s; with a final extension at 72 °C for 5 min. The amplified products were resolved by electrophoresis on 8% polyacrylamide gels.

## 5. Conclusions and Perspectives

This study explored panicle-neck injection of exogenous total DNA as a potential approach for utilizing desirable genes from wild rice and for partially overcoming reproductive barriers. The newly developed restorer line ‘Hai Hui 2011’ and its hybrid ‘Te You 2011’ showed a positive average yield advantage across the tested environments and strong resistance to the predominant Hainan blast population YF under the tested conditions. Future research should focus on: (1) whole-genome resequencing, transcriptomic analysis, and fine mapping of the donor-specific fragments to determine whether donor-DNA integration has occurred and to identify novel genes or regulatory elements; (2) conducting multi-year, multi-location regional trials to evaluate the adaptability and commercial potential of ‘Te You 2011’ across different ecological zones; and (3) using marker-assisted selection to introgress the resistance genes from ‘Hai Hui 2011’ into other elite restorer lines, thereby broadening the genetic base for resistance breeding. This study provides a paradigm for the efficient utilization of wild rice germplasm and has positive implications for addressing the increasing threat of blast disease under global climate warming.

## Figures and Tables

**Figure 1:**
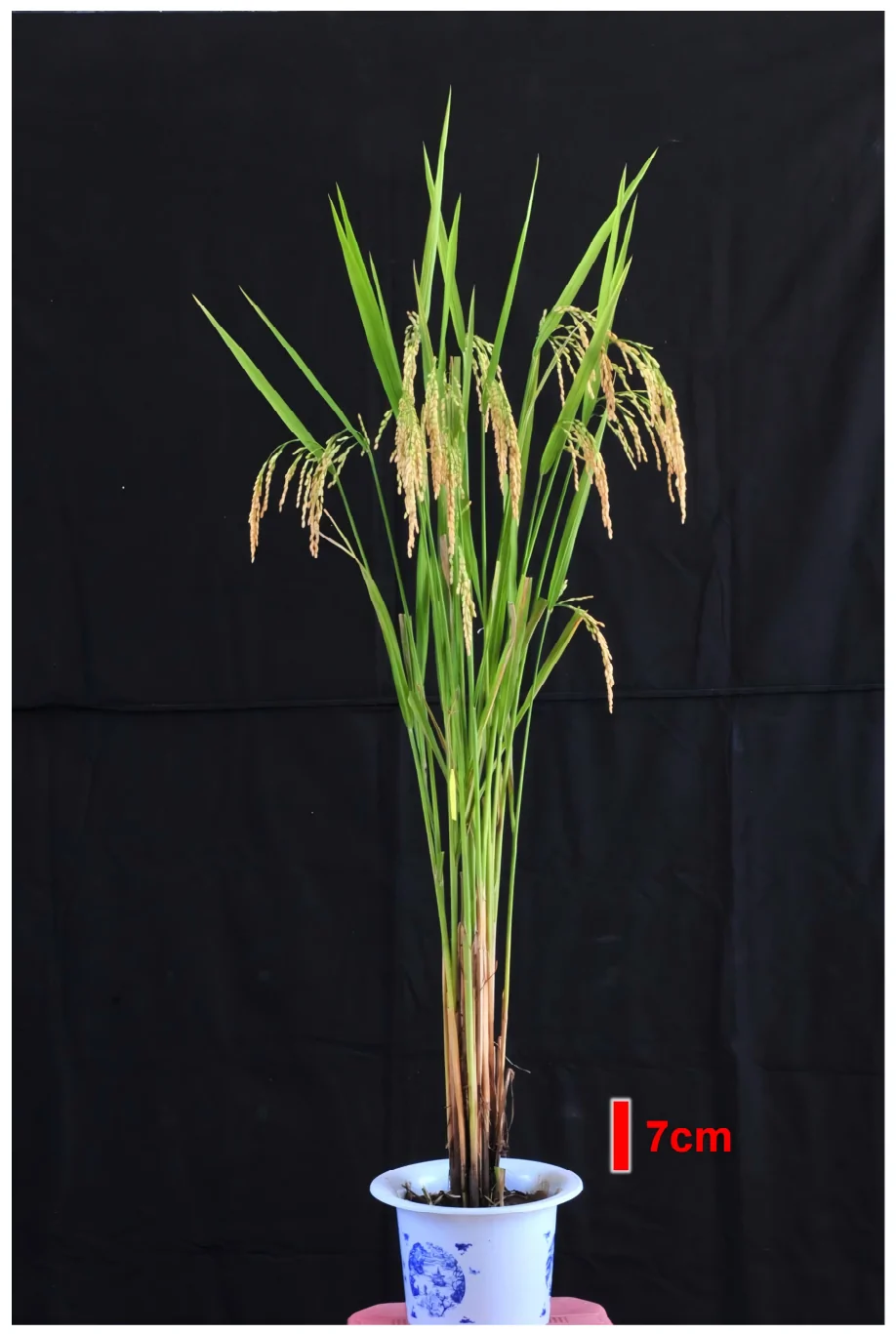
A single plant photo of ‘Hai Hui 2011’.

**Figure 2:**
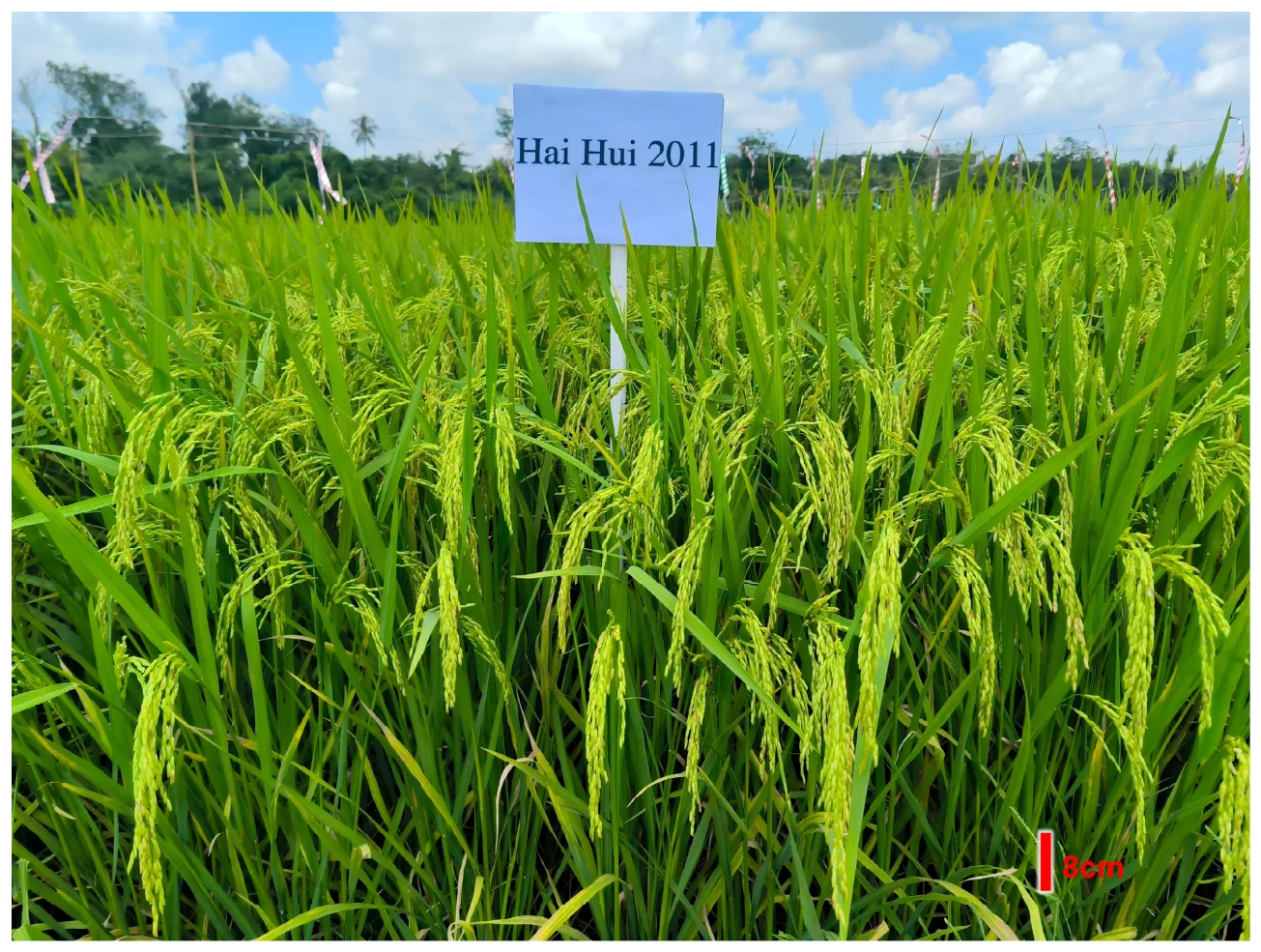
Overall photo of ‘Hai Hui 2011’ growing in a paddy field.

**Figure 3:**
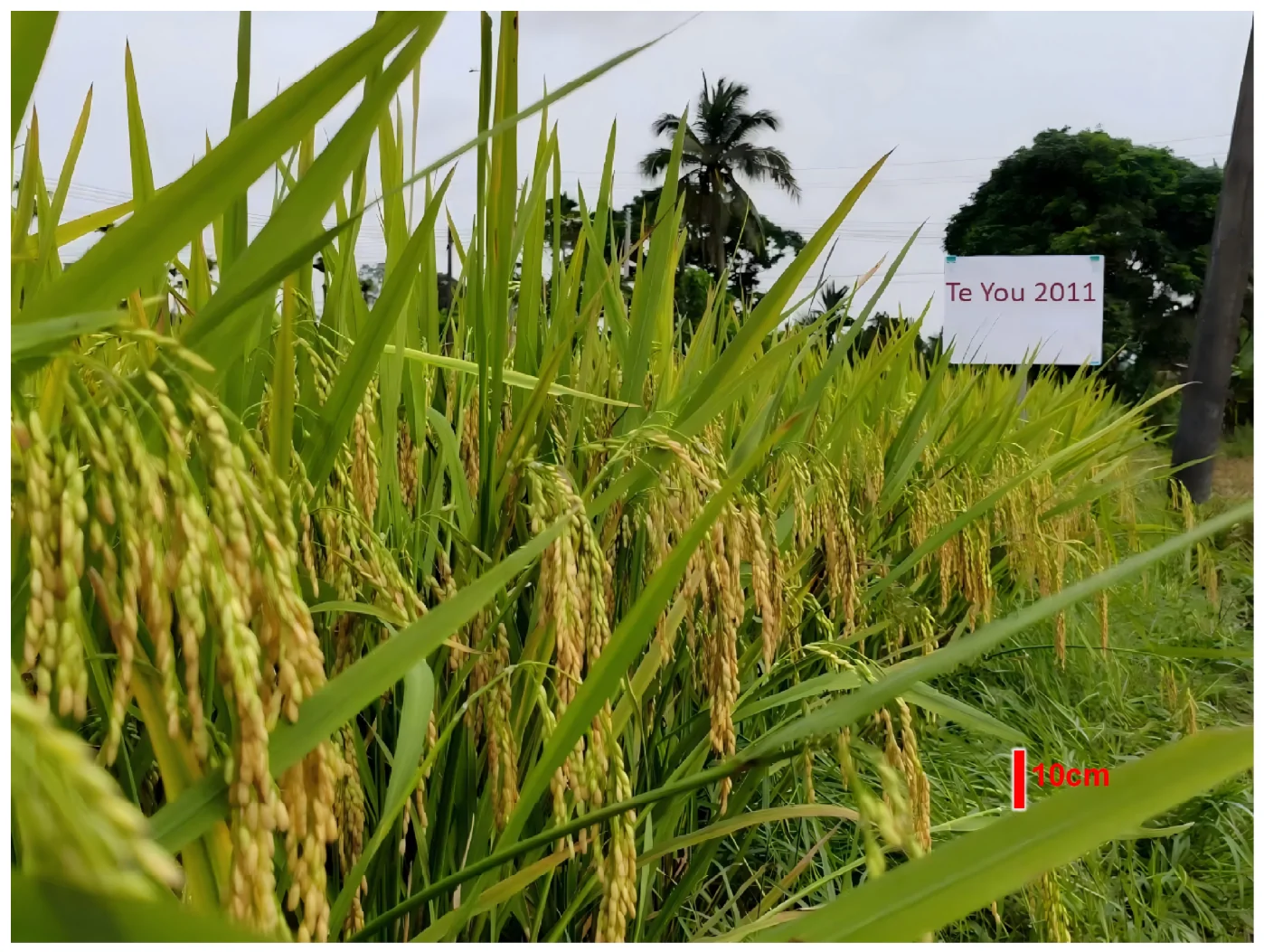
Field view of ‘Te You 2011’.

**Figure 4:**
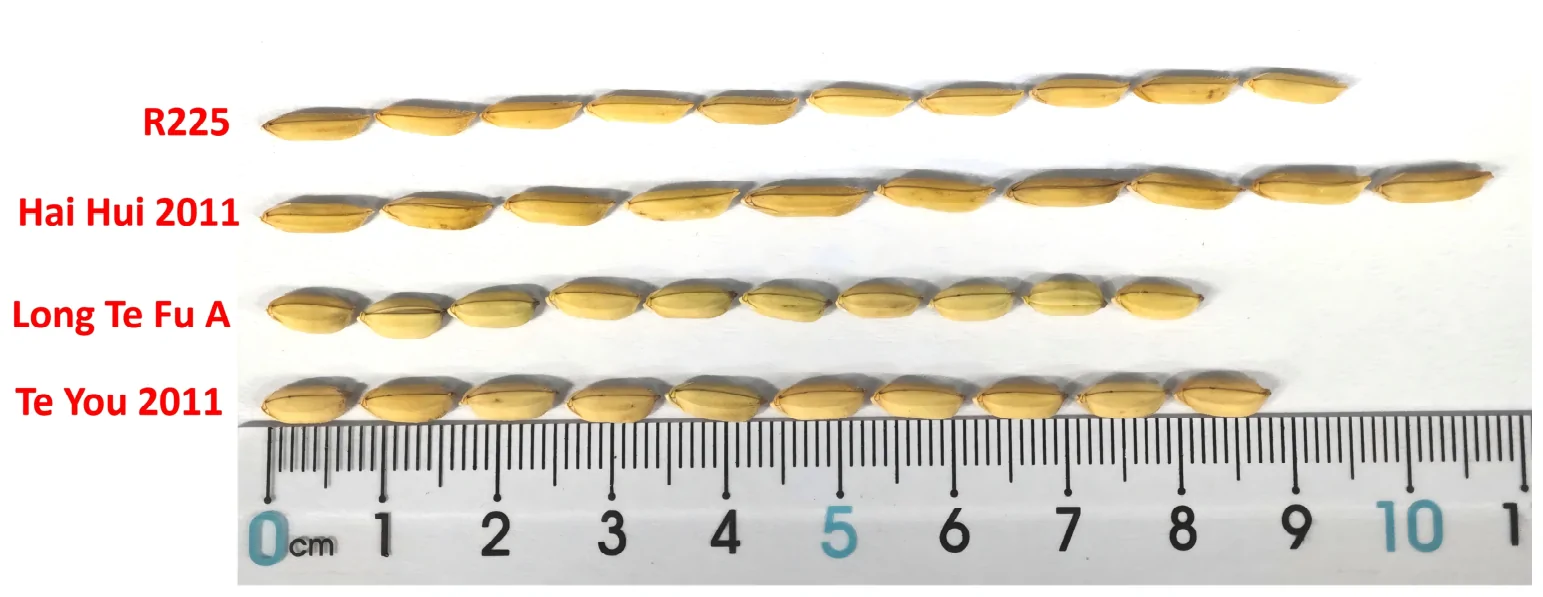
Seed type of R225, Hai Hui 2011, Long Te Fu A, Te You 2011.

**Figure 5:**
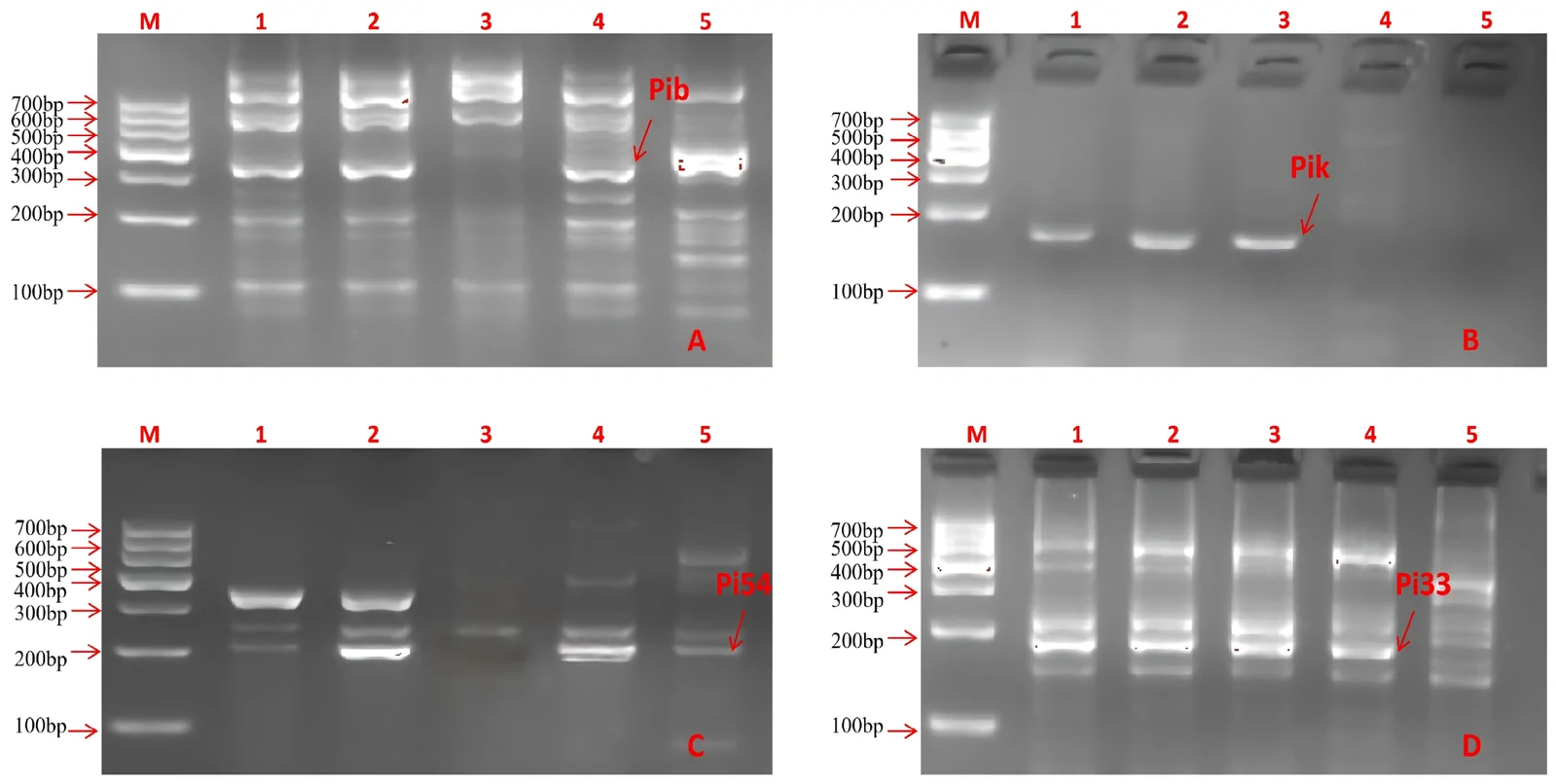
Primer amplification results of rice blast resistance genes. (**A**): Amplification results of the blast resistance gene *Pib*, (**B**): Amplification results of the blast resistance gene *Pik*, (**C**): Amplification results of the blast resistance gene *Pi54*, (**D**): Amplification results of the blast resistance gene *Pi33*, M: DL700 DNA ladder Marker; 1: Hai Hui 2011; 2: Te You 2011; 3: R225; 4: Long Te Fu A; 5: *Oryza officinalis.*

**Figure 6:**
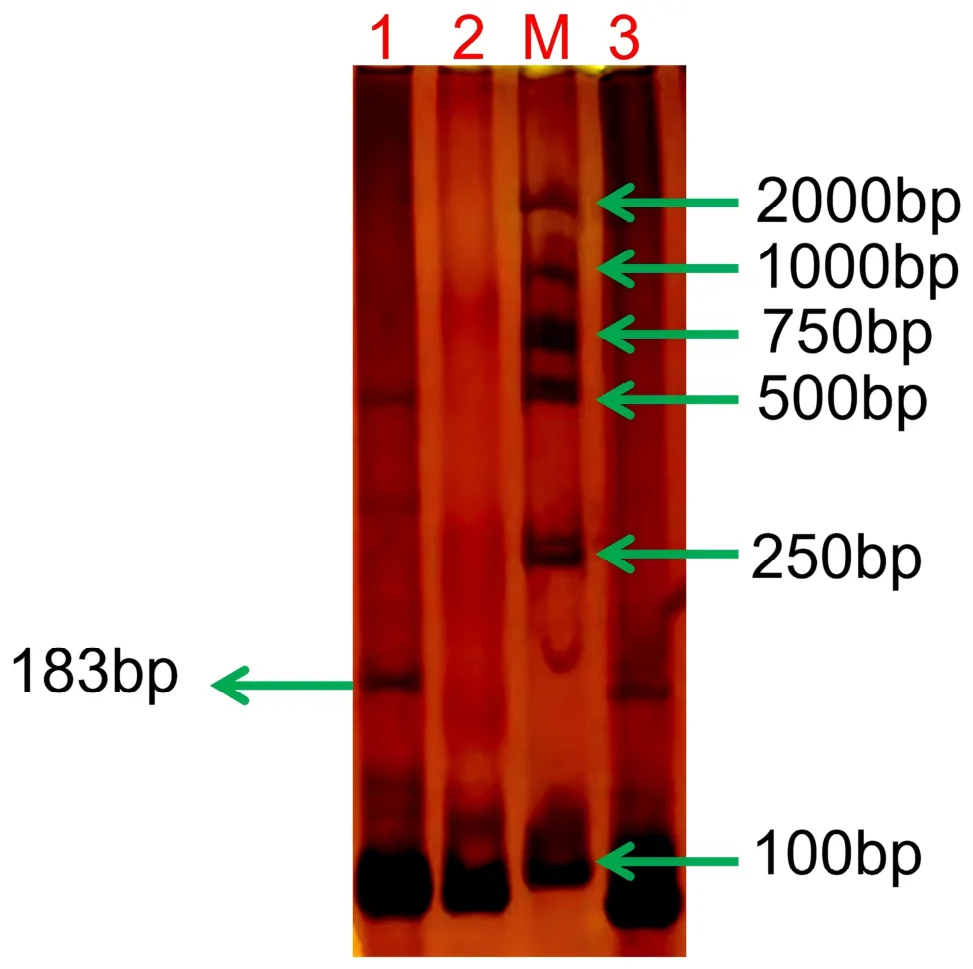
Primer RM154 amplification electrophoresis image. 1: Hai Hui 2011; 2: R225; M: Marker; 3: *Oryza officinalis*.

**Figure 7:**
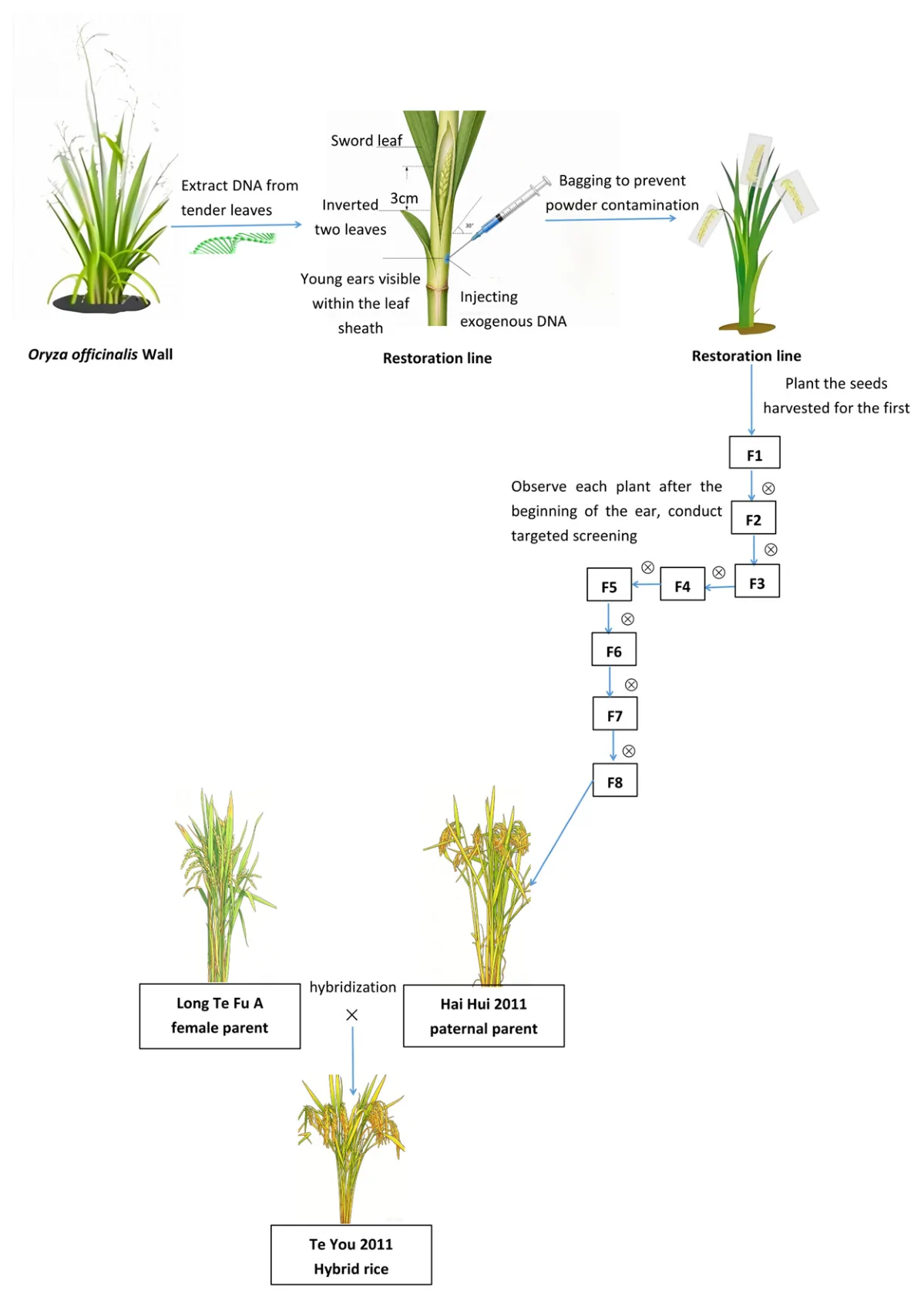
Breeding roadmap for ‘Hai Hui 2011’ and ‘Te You 2011’; ⊗ represents self-pollination; × represents hybridization.

**Table 1:** Restore the Phenotype Data of the Restorer Line ‘Hai Hui 2011’ and Hybrid ‘Te You 2011’.

Agronomic Traits	Growth Period	Plant Height/cm	Panicle Number	Panicle Length/cm	Number of Grains per Panicle	Seed-Setting Rate/%	1000-Grain Weight/g
Hai Hui 2011	Early rice 125 days/late rice 120 days	110.5	7.3	21.3	140.5	85.8	25.3
Te You 2011	Early rice 125–130 days/late rice 115.3 days	108.8	6.2	22.2	153.3	89.8%	28.6

**Table 2:** Regional Trial Yield Results of Early Rice in Hainan Province, 2020–2021.

Time	Trial Site	Te You 2011 (t/ha)	Te You 009 (CK) (t/ha)	Rate of Growth (%)
2020	Chengmai	8.28 ± 0.08	7.92 ± 0.05	4.55
	Sanya	7.70 ± 0.03	7.25 ± 0.06	6.21
	Tunchang	7.72 ± 0.05	7.23 ± 0.08	6.78
	Danzhou	8.25 ± 0.03	7.91 ± 0.04	4.30
	Qionghai	7.75 ± 0.05	7.22 ± 0.06	7.34
	Lingshui	11.72 ± 0.06	10.86 ± 0.05	7.92
	Mean ± (SE)	8.57 ± 0.64	8.07 ± 0.58	6.20 **
2021	Chengmai	8.70 ± 0.06	8.05 ± 0.05	8.07
	Sanya	9.68 ± 0.05	9.00 ± 0.06	7.56
	Tunchang	8.96 ± 0.04	9.48 ± 0.03	−5.48
	Danzhou	8.66 ± 0.07	8.82 ± 0.05	−1.81
	Qionghai	10.71 ± 0.03	10.19 ± 0.06	5.10
	Lingshui	5.46 ± 0.06	5.52 ± 0.07	−1.09
	Mean ± (SE)	8.70 ± 0.72	8.51 ± 0.66	2.23

Note: The asterisk indicates the significance level: ** *p* < 0.01.

**Table 3:** Production Trial Yield Results of Early Rice in Hainan Province, 2021.

Time	Trial Site	Te You 2011 (t/ha)	Te You 009 (CK) (t/ha)	Rate of Growth (%)
2021	Chengmai	8.80 ± 0.02	7.11 ± 0.04	23.77
	Sanya	8.85 ± 0.01	8.75 ± 0.02	1.14
	Tunchang	8.40 ± 0.03	9.00 ± 0.03	−6.67
	Danzhou	8.46 ± 0.06	7.88 ± 0.04	7.36
	Qionghai	5.67 ± 0.03	5.10 ± 0.06	11.18
	Lingshui	10.72 ± 0.05	8.56 ± 0.02	25.23
Mean ± (SE)		8.48 ± 0.66	7.73 ± 0.60	9.70

**Table 4:** Independent Expanded Season Trial Yield Results of Late Rice, 2024.

Time	Trial Site	Te You 2011 (t/ha)	Te You 009 (CK) (t/ha)	Rate of Growth (%)
2024	Hainan Qionghai	4.66 ± 0.06	4.24 ± 0.03	9.91
	Hainan Wenchang	5.94 ± 0.04	5.97 ± 0.05	−0.50
	Hainan Ding’an	6.09 ± 0.05	6.01 ± 0.02	1.33
	Hainan Danzhou	6.51 ± 0.02	6.16 ± 0.05	5.68
	Hainan Changjiang	6.19 ± 0.03	4.78 ± 0.06	29.50
	Hainan Wuzhishan	6.04 ± 0.01	5.83 ± 0.02	3.60
	Hainan Sanya	6.26 ± 0.06	5.50 ± 0.03	13.82
Mean ± (SE)		5.96 ± 0.23	5.50 ± 0.27	8.36 *

Note: The asterisk indicates the significance level: * *p* < 0.05.

**Table 5:** Molecular marker information for identification of resistance genes to rice blast disease.

Primer	PCR Product Size (bp)	Primer Sequence (5′–3′)		Reference
Pia	189	GCGACTGACACTTTCAATAGC	CGGTAGAGCAATTTAGAAGCAG	[22]
Pib	365	GAACAATGCCCAAACTTGAGA	GGGTCCACATGTCAGTGAGC	[23]
Pi33	198	AGCTGACCAACCAGGCGATG	TGCAACATTCGATCCAACCC	[20]
Pid2	233	GGCTCGATCTAGAAAATCCG	TTGCACAGGTTGCGATAGAG	[24]
Pik	176	TTCGTTTTCCTTGGTTAGTG	ATTGGCTCCTGAAGAAGG	[25]
Pigm	236	ATGCTCGATTCGTTACATTT	CGAGTGCTTTTATAGCGAAT	[26]
Pi21	350	GGAGATCCTCATCGTCGACGT	CGGAGCCACCGAAGGAGCC	[27]
Pi9	291	GCTGTGCTCCAAATGAGGAT	GCGATCTCACATCCTTTGCT	[28]
Pi20	210	GCTGAGGAGTATCCTTTCTC	ACCATAGGAAGATCATCACA	[29,30]
Pi54	216	CAATCTCCAAAGTTTTCAGG	GCTTCAATCACTGCTAGACC	[31]

**Table 6:** Thirty-three pairs of SSR primers used to preliminarily search the genetic relationship between *O. officinalis* and ‘Hai Hui 2011’.

Primer	Primer Sequence (5′–3′)	
RM583	AGATCCATCCCTGTGGAGAG	GCGAACTCGCGTTGTAATC
RM443	GATGGTTTTCATCGGCTACG	AGTCCCAGAATGTCGTTTCG
RM212	CCACTTTCAGCTACTACCAG	CACCCATTTGTCTCTCATTATG
RM220	GGAAGGTAACTGTTTCCAAC	GAAATGCTTCCCACATGTCT
RM135	CTCTGTCTCCTCCCCCGCGTCG	TCAGCTTCTGGCCGGCCTCCTC
RM282	CTGTGTCGAAAGGCTGCAC	CAGTCCTGTGTTGCAGCAAG
RM168	TGCTGCTTGCCTGCTTCCTTT	GAAACGAATCAATCCACGGC
RM231	CCAGATTATTTCCTGAGGTC	CACTTGCATAGTTCTGCATTG
RM3131	CTCTGCACCCTGTTCACATG	CCCAATGGAATATCAGGTGG
RM498	AATCTGGGCCTGCTCTTTTC	TCCTAGGGTGAAGAAAGGGG
RM154	ACCCTCTCCGCCTCGCCTCCTC	CTCCTCCTCCTGCGACCGCTCC
RM318	GTACGGAAAACATGGTAGGAAG	TCGAGGGAAGGATCTGGTC
RM1694	CCCTATCTTTAGATCCCAATGTA	CATGGAGCACCAAACAAAT
RM485	CACACTTTCCAGTCCTCTCC	CATCTTCCTCTCTTCGGCAC
RM2326	AGTCCCCTAGTCAATATCAT	ATAGGCAACACATTGTACAT
RM131	TCCTCCCTCCCTTCGCCCACTG	CGATGTTCGCCATGGCTGCTCC
RM537	CCGTCCCTCTCTCTCCTTTC	ACAGGGAAACCATCCTCCTC
RM3042	CAAAAAGGAATCAATGTGAA	GGCTGTTGAGAGGTAGAGAA
RM551	AGCCCAGACTAGCATGATTG	GAAGGCGAGAAGGATCACAG
RM118	CCAATCGGAGCCACCGGAGAGC	CACATCCTCCAGCGACGCCGAG
RM538	GGTCGTTGAAGCTTACCAGC	ACAAGCTCTCAAAACTCGCC
RM5796	GCGATGGAACATGAAGTGTG	TGGATGTTCTGATGCAGAGC
RM6728	GGGTATGTGTCGCTATTTTA	GAAATCTGGAATTTTCCCTA
RM549	ACGAACTGATCATATCCGCC	CTGTGGTTGATCCCTGAACC
RM548	TCGGTGAGAAACTGAGAGTACG	AAGGAGGCCATCTCAATGTG
RM542	TGAATCAAGCCCCTCACTAC	CTGCAACGAGTAAGGCAGAG
RM5531	TTTGTGTTGGTAAGTTGCTTC	TTAAGGAGAGTGTTTTCTTTTCTC
RM586	ACCTCGCGTTATTAGGTACCC	GAGATACGCCAACGAGATACC
RM253	TCCTTCAAGAGTGCAAAACC	GCATTGTCATGTCGAAGCC
RM42	ATCCTACCGCTGACCATGAG	TTTGGTCTACGTGGCGTACA
RM6872	GGATGAACACTGATGATGGC	ACCTCCACCACGATATCCAC
RM336	CTTACAGAGAAACGGCATCG	GCTGGTTTGTTTCAGGTTCG
RM18	TTCCCTCTCATGAGCTCCAT	GAGTGCCTGGCGCTGTAC

## Data Availability

The original contributions presented in the study are included in the article; further inquiries can be directed to the corresponding author.
